# Polio outbreaks in Cameroon following the COVID-19 pandemic

**DOI:** 10.11604/pamj.2023.45.90.35332

**Published:** 2023-06-21

**Authors:** Andreas Ateke Njoh, Tchokfe Shalom Ndoula Josue Kedakse, Ekoum Eric Mboke, Raoul Nembot, Lele Youmssi Parfait Collins, Adidja Amani, Hassan Ben Bachire, Kabiru Abubakar Gulma, Laurent Cleenewerck De Kiev

**Affiliations:** 1 Expanded Program on Immunization, Ministry of Public Health, Yaoundé, Cameroon,; 2 School of Global Health and Bioethics, Euclid University, Bangui, Central African Republic,; 3 World Health Organization, Cameroon Country Office, Yaoundé, Cameroon,; 4 Ministry of Public Health, Yaoundé, Cameroon

**Keywords:** Polio, outbreak, circulating vaccine-derived poliovirus, vaccine, COVID-19, Cameroon

## Abstract

Polio is an infectious and disabling life-threatening disease caused by the poliovirus. This disease is prevented through vaccination. Though this viral infection has been eliminated in most parts of the world, a few countries are still endemic to wild poliovirus. In 2020, the World Health Organization (WHO) African Region, including Cameroon, was certified free of wild poliovirus. Some countries recurrently report circulating vaccine-derived poliovirus cases (cVDPV) despite recorded achievements. Also, the risk of importing poliovirus from endemic settings remains, particularly in the context of coronavirus disease (COVID-19). This study aimed to assess the state of polio in Cameroon and identify the situation during COVID-19. A data review was conducted from February to March 2023. Data on polio cases and vaccination coverage per region of Cameroon were reviewed from 2014 to 2022. Data were analyzed with Microsoft Excel, and the results were presented as proportions. The last wild poliovirus was reported in Cameroon in 2014, and the country benefitted from a response. No case of poliovirus was detected in the country from 2015 to 2018. After that, an increasing number of type two cVDPV were reported across 50% of the country's regions from 2019 to 2022. The outbreaks benefitted from responses with various oral polio vaccines, including the type two novel oral polio vaccine (nOPV-2). Though wild polioviruses have been eliminated in most countries, including Cameroon, cVDPV remains a significant problem. There is an urgent need to strengthen disease surveillance and vaccination to prevent cVDPV-2 in this country, particularly in the COVID-19 context.

## Introduction

Many countries are reporting increasing polio activity recently, though most continents have eliminated wild polioviruses. Poliomyelitis (polio) is a highly infectious viral disease that affects humans. The virus predominantly infects infants under five years, and it is responsible for paralysis, and in a few cases, it can lead to death. However, most persons infected with the virus do not develop the disease. Notwithstanding, less than 25% of infected persons develop flulike symptoms, while less than 1% of cases develop a more severe illness with paralysis. In some cases, children who fully recover from poliomyelitis develop paralysis years later (7 to 15 years) as adults [1].

There are currently three known serotypes of wild polioviruses (type 1, type 2, and type 3). Infection is mainly through fecal-oral contamination and sometimes oral-oral [2]. Following infection, the virus can replicate in the oropharyngeal and intestinal lymphatics [2]. In unimmunized or under-immunized persons, the virus could spread to the central nervous system and cause anterior horn neuronal death, resulting in pure motor deficits [3].

Polio vaccines were first introduced in the 1950s. This vaccine introduction followed the discovery of the Salk trivalent inactivated polio vaccine in 1956, which was administered intramuscularly or subcutaneously. This progress led to a significant reduction in polio cases. Since this trivalent vaccine does not stimulate secretory immunoglobulin A, it was soon replaced by Sabin trivalent oral polio vaccine (tOPV) in 1962. The Sabin trivalent OPV contains live attenuated strains of polioviruses type 1, 2, and 3. This trivalent vaccine provides both serum antibodies and local gastrointestinal antibodies. In addition, it leads to viral excretion from OPV that favors community infection and can lead to herd immunity [3].

To protect from poliomyelitis, the population must have immunity against all three forms of poliovirus. Vaccination has been the most effective way to protect against polio. There are two primary polio vaccine types: oral polio (OPV) and inactivated polio (IPV) vaccine. Each type of OPV contains at least one, two, or all three types of live attenuated polioviruses; administered per os. Meanwhile, the IPV has all three inactivated polioviruses; administered intramuscularly.

The OPV is the primary vaccine used in the fight against poliomyelitis. The attenuated poliovirus in OPV replicates in the intestine but is less likely to enter the nervous system. OPV provides long-lasting and efficient protection against the polioviruses that they target. The replicated OPV could be shed in the community several weeks after administration. Though the OPV vaccine is very effective, it could cause paralytic disease in rare cases [4]. Due to the risk of poliomyelitis due to OPV, there was a global request to remove type 2 attenuated virus from OPV and scale up the use of IPV [2]. Inactivated polio(IPV) stimulates the immune system to protect against all three poliovirus strains. Since IPV contains inactivated viral strains, there is no risk of viral reactivation and community spread from vaccinated persons. However, persons vaccinated with IPV can still be infected with wild polioviruses, and they shed the virus in the community. Though IPV confers a lower level of immunity in the intestine, it efficiently protects against paralytic diseases due to poliovirus [5].

If a population is under-immunized, some children could get infected by the vaccine-derived polioviruses (VDPV) shed by vaccinated persons. This virus in circulation can mutate after over a year and become neurovirulent. This new virus is called circulating vaccine-derived polioviruses (cVDPV). However, a fully immunized population is protected from cVDPV [6]. With the challenge associated with cVDPV, researchers developed a novel type-2 oral polio vaccine (nOPV-2) following modifications in the Sabin type 2 OPV strain genome commonly responsible for most cVDPV-2. This genetic modification makes the nOPV-2 strain stable and unlikely to form cVDPV [7]. This discovery has motivated the large-scale use of nOPV-2, particularly in the control of cVDPV-2.

Despite the achievement in polio control, the disease has remained a significant public health problem. This problem motivated the creation of the Global Polio Eradication Initiative (GPEI) in 1988. The GPEI interventions have brought polio under control globally. Though wild polio has been eradicated in most parts of the world, including Cameroon, countries like Afghanistan and Pakistan are still endemic to the virus with the risk of exportation to every end of the globe [8]. Many African countries have reported increasing cVDPV for a couple of years. In early 2022, cases of wild poliovirus were reported in Malawi and Mozambique [9,10].

Cameroon maintains a resilient fight against polioviruses after controlling its last wild poliovirus in 2014 [11]. Various vaccine forms have permitted the health system to limit polio spread. The Expanded Program on Immunization (EPI) initially used trivalent OPV (tOPV) but later switched to using the bivalent OPV (bOPV) and the IPV for routine immunization. The bOPV is routinely used during annual mass campaigns to control the disease in the Country. In addition, various monovalent OPV (mOPV) have been used in parts of the country to control outbreaks. The country has progressively improved its vaccination coverage against the virus in the past three decades. Disease surveillance has been strengthened to meet up with the goals of the GPEI. With the achievements in the polio fight, the country was certified free of wild poliovirus in 2020 [12]. Despite the polio-free status, polio remains a global health problem, and Cameroon's Ministry of Health maintains its efforts to ensure a robust polio response.

Like the rest of the world, Cameroon was hit by the coronavirus disease (COVID)-19 in early 2020, with a progressive increase in cases and deaths across all regions. The COVID-19 control measures associated with vaccine hesitancy exacerbated by the introduction of the COVID-19 vaccine contributed to the fall in the use of health services and a drop in vaccination coverage of childhood vaccines [13]. Following this observation, we evaluated polio in Cameroon before and following COVID-19. We reviewed the trend in vaccination coverage and polio cases over the years. We also looked at the site of the outbreak and the lineage of the viruses reported, and the vaccination responses.

## Methods

Following the increasing polio cases reported worldwide in recent days and the impact of COVID-19, this study aimed to review poliovirus and vaccination activities in Cameroon from 2014 to 2022 to have a clear picture of the evolution of poliovirus and vaccination before Cameroon's polio-free status and three years into the COVID-19 context. This study period helps to depict the evolution of the problem since the last wild poliovirus was reported in the country. The timeframe also captures the polio-free status 2020 certification year and COVID-19 up to 2022.

**Study setting:** this study was carried out in Cameroon. According to the national population estimate for 2022, the country had a total population of 27,795,843, with 25% aged less than 15 years. This country is divided into ten regions, with four regions (Far North, North West, South West, and East) experiencing insecurity and periodic mass population movements [14]. Cameroon is one of the countries of the Lake Chad Basin, and it experiences population movement across its borders with the neighboring countries. Like the rest of Africa, this country was certified free of wild poliovirus in June 2020 [15].

**Study design and data sources:** we conducted a data review from February to March 2023. Data on polio cases and vaccination coverage per region of Cameroon were reviewed over the past nine years (2014 to 2022). Polio surveillance data were obtained from Cameroon's weekly epidemiological reports of the EPI. The data on the virus lineage in the EPI weekly report was an image report in the surveillance database of the WHO Regional Office shared with Cameroons EPI.

Routine immunization data were obtained from the district health information software (Dhis)-2 from 2017 to 2022. Unfortunately, the system did not capture vaccination data before this time. Data on polio response campaigns carried out during the study period were abstracted from the Central Technical Group annual reports for EPI. The quality of vaccination campaigns was reported as indicated in the EPI database. The campaign quality considered the proportion of the target vaccinators missed. During lot quality assessment, if the independent monitors find that vaccinators missed at least three children randomly selected, they consider the vaccination poor in that community.

**Variables and measurement:** vaccination coverage accounted for the number of infants vaccinated per vaccine type expressed as a proportion of the target population per region per year. The lineage of the poliovirus in Cameroon was obtained as reported in the database of the EPI. This information was an element reported by the EPI that reflects the WHO regional office database shared with the country. The lineage report considered nucleotide similarities between the identified varus and previously found cVDPV in other countries. The site of identification of poliovirus was based on the geolocation reported by the health staff in the open data kit (ODK) as the residence of the person from whose stool the poliovirus was isolated or the location of the environmental sample collection site.

**Data management and analysis:** the data were analyzed with Microsoft Office Excel 2019, and summary statistics were used to estimate vaccination coverage. Geocoordinates were presented in Excel and uploaded to the quantum geoinformation system (QGIS) to report the data on maps.

This review was a secondary study that used data abstracted from the existing data of the Ministry of Health. So, no ethics approval and consent to participate were required.

## Results

**Evolution of routine vaccination coverage:** over the past six years, the polio vaccination coverage at the national level has remained less than 80%. Before 2020, sixty percent of the regions had a polio vaccination coverage of at least 80%. However, from 2020 the proportion of the country's regions with at least eighty percent polio vaccination coverage dropped to (4/10 regions) 40% (Table 1, Table 2).

**Table 1:** routine vaccination coverage of inactivated polio vaccine (IPV) 2017-2022

Regions	IPV					
	2017	2018	2019	2020	2021	2022
Adamawa	82%^#^	82%^#^	88%^#^	91%^#^	88%^#^	90.3%^#^
Centre	75%&	90%^#^	94%^#^	88%^#^	81%^#^	82%^#^
East	87%^#^	94%^#^	102%^#^	99%^#^	91%^#^	100.7%^#^
Far North	69%&	73%&	74%&	77%&	81%^#^	77.1%&
Littoral	80%^#^	84%^#^	81%^#^	78%&	70%&	74.9%&
North	76%&	81%^#^	85%^#^	95%^#^	90%^#^	89.5%^#^
Northwest	78%&	57%&	48%*	55%&	72%&	72%&
West	71%&	78%&	73%&	75%&	70%&	73.4%&
South	93%^#^	92%^#^	86%^#^	74%&	73%&	80.2%^#^
Southwest	89%^#^	54%&	47%*	68%&	66%&	63.1%&
Cameroon	76.3%&	77.7%&	77.8%&	79%&	78.9%&	79.8%&

* : indicating the regions with less than 50% vaccination coverage for the corresponding period; &: indicate the regions with vaccination coverage from 50% and 79% for the corresponding period; ^#^: depict the regions with vaccination overage of 80% and above for the corresponding period

**Table 2:** routine vaccination coverage of oral polio vaccine (OPV) 2017-2022

Regions	OPV3					
	2017	2018	2019	2020	2021	2022
Adamawa	83%^&^	83%^&^	88%^&^	90%^&^	87%^&^	82%^&^
Centre	94%^&^	95%^&^	95%^&^	87%^&^	81%	73%^#^
East	98%^&^	99%^&^	104%^&^	101%^&^	92%^&^	97%^&^
Far North	75%^#^	72%^#^	75%^#^	77%^#^	82%^&^	58%^#^
Littoral	87%^&^	85%^&^	81%	78%^#^	70%^#^	60%^#^
North	81%^&^	80%^&^	84%^&^	93%^&^	88%^&^	81%^&^
Northwest	78%^#^	57%^#^	49%^*^	56%^#^	73%^#^	69%^#^
West	81%^&^	75%^#^	73%^#^	71%^#^	70%^#^	69%^#^
South	97%^&^	90%^&^	86%^&^	75%^#^	72%^#^	63%^#^
Southwest	90%^&^	54%^#^	47%^*^	71%^#^	67%^#^	62%^#^
Cameroon	83.8%^&^	78.1%^#^	78.2%^#^	79.8%^#^	79.1%^#^	69%^#^

* : regions with less than 50% vaccination coverage for the corresponding period; ^#^: indicate the regions with vaccination coverage from 50% and 79% for the corresponding period; ^&^: depict the regions with vaccination coverage of 80% and above for the corresponding period; OPV: oral polio vaccine

**Figure 1:**
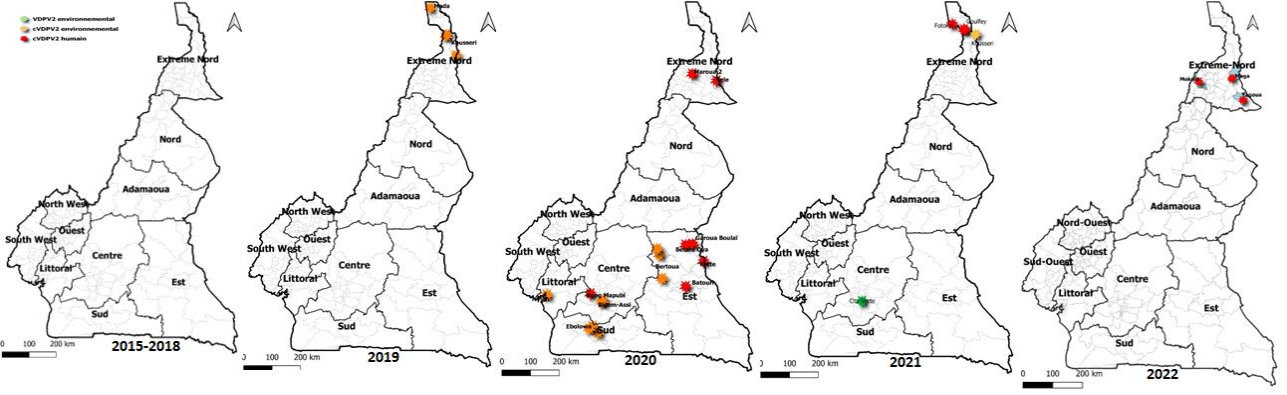
type 2 vaccine-derived polioviruses (VDPV) and type-2 cVDPV cases reported from 2015 to 2022

**Evolution of polio cases:** after controlling the last case of wild poliovirus in 2014 in Cameroon, surveillance was reinforced. Until 2018, though acute flaccid paralysis (AFP) cases were investigated in all of the country's ten regions and environmental samples analyzed, wild poliovirus or cVDPV was not isolated (Figure 1). In 2019, cVDPV-2 in the Far North Region was identified in environmental samples. In 2020, cVDPV-2 was identified in human and environmental samples in 50% of the country's ten regions (Figure 1). By 2021, more cases of cVDPV-2 were identified in both human and environmental samples in the Far North. In the second half of 2022, cVDPV-2 was identified in the stool of two infants presenting acute flaccid paralysis (AFP) and a contact (Figure 1). Two of the infants had received the nOPV vaccine during the previous campaign rounds.

**The lineage of polio cases:** all cVDPV identified from 2019 to 2022 had similarities to initially identified viruses from neighboring countries, including Nigeria, Chad, and the Central African Republic (Table 3). In 2021, environmental samples from two sites in the Center Region of Cameroon presented with VDPV-2, which was not similar to those from other locations.

**Table 3:** vaccine-derived polioviruses (VDPV) type-2 and circulating vaccine-derived polioviruses (cVDPV) type-2 from 2017-2022 in Cameroon

Year of sampling	Region of sample collection	Virus category	Source of specimen	VDPV Nucleotide difference from SABIN 2	Date of sample collection	Lineage
	Far North	cVDPV2	Environment	20	20-Apr-19	Nigeria
	Far North	cVDPV2	Environment	10	21-Oct-19	Chad
2019	Far North	cVDPV2	Environment	30	2-Dec-19	
	Far North	cVDPV2	Environment	7	16-Dec-19	
	East	cVDPV2	Infant	18	14-Feb-20	Central Africa Republic
	Littoral	cVDPV2	Infant	13	29-Feb-20	Chad
	East	cVDPV2	Environment	13	2-Mar-20	
	East	cVDPV2	Infant	15	11-Apr-20	Central Africa Republic
	East	cVDPV2	Environment	19	5-May-20	
	East	cVDPV2	Environment	19	2-Jun-20	Nigeria
	East	cVDPV2	Infant	19	15-Jul-20	Central Africa Republic
	Littoral	cVDPV 2	Environment	20	29-Jul-20	
	South	cVDPV2	Environment	19	26-Aug-20	
	Center	cVDPV2	Environment	20	1-Sep-20	
2020	South	cVDPV2	Environment	20	3-Sep-20	
	Center	cVDPV2	Environment	14	6-Sep-20	
	Far North	cVDPV2	Infant	15	11-Sep-20	Chad
	South	cVDPV2	Environment	21	29-Sep-20	Central Africa Republic
	Center	VDPV2	Environment	6	16-Nov-20	Unknown
2021	Center	VDPV2	Environment	7	11-Jan-21	
	Far North	cVDPV2	Infant	24	1-Sep-21	Nigeria
	Far North	cVDPV2	Infant	23	21-Sep-21	
	Far North	cVDPV2	Infant	26	11-Oct-21	
	Far North	cVDPV2	Environment	24	25-Oct-21	
2022	Far North	cVDPV2	Infant	38	2-Oct-2022	Nigeria
	Far North	cVDPV2	Infant	38	3-Nov-2022	
	Far North	cVDPV2	Infant	41	31-Dec-2022	

**Response campaigns against cVDPV:** the 2019 outbreak regions benefitted from two-round vaccination responses with mOPV in six regions. The vaccination coverage for people 0 to 59 months was 86.6% and 92.5%. At least 51 (44%) out of 166 districts involved were rejected in each round during lot quality assurance sampling (LQAS) for poor execution of the vaccination campaign after independent monitoring. In 2020, due to the COVID-19 restrictions, the response was delayed. However, a round of nationwide vaccination was carried out with the bivalent Oral Polio Vaccine (bOPV) in May, with a coverage of 87.1% for people 0 to 59 months. At least 59 (33%) out of 181 health districts involved were rejected during LQAS for poor execution of the vaccination campaign.

In 2021, a round of nationwide vaccination was carried out in May with a coverage of 87.1% for infants 0 to 59 months. At least 107 (59%) out of 181 health districts involved were rejected during LQAS for sub-optimal quality campaigns following independent monitoring.

In 2022, with the availability of nOPV-2, the country had two nationwide vaccination campaigns for infants 0 to 59 months in May and July, with coverages of 95% and 101%, respectively. At least 75 (40%) out of 189 health districts involved were rejected during LQAS by the evaluators for sub-optimal coverage by independent monitors. As a result of the poor performance, the health actors conducted a third round in November in 41 districts of three regions (Far North, Littoral, and West) with the poorest performances in the previous sessions. The vaccination coverage was 106.2%. At least 21 (51%) out of 41 health districts involved were rejected during LQAS by the independent evaluators for low-quality vaccination coverage.

## Discussion

Cameroon made significant progress in strengthening disease surveillance and polio immunization in the quest to eradicate wild poliovirus within the past decades. By 2014, the country controlled its last wild poliovirus. Before 2019, though acute flaccid paralysis (AFP) cases and environmental samples were investigated, no poliovirus case was detected. However, in 2019, cases of cVDPV with nucleotide similarity to viruses from neighboring African countries were reported in the country. Like the rest of the world, Cameroon suffered from the burden of COVID-19 on immunization [16]. With the COVID-19 burden on health systems, we decided to evaluate the trend in polioviruses in Cameroon. The aim was to review poliovirus and vaccination activities in Cameroon from 2014 to 2022 to have a clear picture of the evolution of poliovirus and vaccination before and three years into the COVID-19 context.

With the emergence of cVDPV in Cameroon in 2019, the country benefitted from mass vaccination with the mOPV. In addition, with the COVID-19 outbreak, the proportion of regions with suboptimal polio vaccination coverage in routine immunization continued to increase. Unfortunately, low immunization coverage significantly exposes the population to epidemics [17,18]. Despite the response campaigns with the mOPV, more cases of cVDPV have been detected, touching more regions of the country over the years (Figure 1). Worse still, there is free circulation in Cameroon and with other countries in the African Region. So, the risk of transporting polioviruses from one zone to another remains.

In addition, bOPV is the primary vaccine used in routine immunization, while mOPV-2 was used in response to the cVDPV outbreaks across the country in the past. Though vaccination remains highly effective in protecting against polio, the risk of circulating poliovirus from vaccinated infants remains [19]. This risk of circulating virus is higher in communities with suboptimal vaccination coverage [6]. We note that the country has persisted with suboptimal vaccination coverage for several years. This coverage worsened in 2020 when COVID-19 was first reported. The worsening vaccination coverage follows vaccine hesitancy marked by the introduction of the COVID-19 vaccination in Cameroon [20]. In addition, persisting insecurity in parts of the country has also contributed to the low vaccination coverage recorded in the country [21].

With the development of the nOPV-2 vaccine, it is believed that the risk of the development of cVDPV-2 is under control since the genetically modified viral form is more stable [7]. Unfortunately, since the last cVDPV outbreak was recorded in 2021, over six months later, it was impossible to respond to this outbreak adequately. This delay increases the risk of the spread of the virus. One of the reasons for the delay in response was the shortage of nOPV on the global stage. Also, more interest is given to countries with a higher outbreak burden. Another reason is that some countries, like Cameroon, did not meet the readiness requirement to use nOPV in the early weeks of the 2021 outbreak [22]. With population movements across the country, this delay in response puts the population at a high risk of a wide-scale spread of poliovirus. In addition, the country organized various response vaccination campaigns with either bOPV, mOPV, or nOPV. Unfortunately, the quality of vaccination remained sub-optimal, as at least 30% of the health districts were rejected for poor vaccination coverage in each round. This factor leaves the target population vulnerable to polioviruses.

This research is the first in Cameroon to elucidate the poliovirus trend following the COVID-19 pandemic. Despite this merit, the assessment of polioviruses in the community was limited to reported information in the Expanded Program on Immunization (EPI) database. This approach could have missed cases that were not captured by the current system. In addition, our study design could not identify the specific factors favoring the rise in vaccine-derived poliovirus cases in this country. Also, the denominators used in all calculations of vaccination coverages were based on national estimates, which may not necessarily reflect the actual situation in the field. There is a need to assess the specific reason for the rise in cVDPV in this country, which was declared free of wild poliovirus by the World Health Organization in 2020.

## Conclusion

In recent years, the world has been experiencing a surge in polio cases after several endemic nations were certified free of wild poliovirus. With the advent of COVID-19, Cameroon's health system was affected. During this period, we observe a fall in coverage and an upward trend in vaccine-derived poliovirus cases in human and environmental samples in most of the country's regions, particularly from 2020. There is, therefore, a need to strengthen vaccination interventions and disease surveillance and rapidly and adequately respond to these outbreaks to prevent further polio spread.
